# Modeling Physico-Chemical ADMET Endpoints with Multitask Graph Convolutional Networks

**DOI:** 10.3390/molecules25010044

**Published:** 2019-12-21

**Authors:** Floriane Montanari, Lara Kuhnke, Antonius Ter Laak, Djork-Arné Clevert

**Affiliations:** Digital Technologies, Bayer AG, 13353 Berlin, Germany; lara.kuhnke@bayer.com (L.K.); antoniuster.laak@bayer.com (A.T.L.); djork-arne.clevert@bayer.com (D.-A.C.)

**Keywords:** ADMET prediction, multitask learning, graph convolutional networks, solubility, QSAR

## Abstract

Simple physico-chemical properties, like logD, solubility, or melting point, can reveal a great deal about how a compound under development might later behave. These data are typically measured for most compounds in drug discovery projects in a medium throughput fashion. Collecting and assembling all the Bayer in-house data related to these properties allowed us to apply powerful machine learning techniques to predict the outcome of those assays for new compounds. In this paper, we report our finding that, especially for predicting physicochemical ADMET endpoints, a multitask graph convolutional approach appears a highly competitive choice. For seven endpoints of interest, we compared the performance of that approach to fully connected neural networks and different single task models. The new model shows increased predictive performance compared to previous modeling methods and will allow early prioritization of compounds even before they are synthesized. In addition, our model follows the generalized solubility equation without being explicitly trained under this constraint.

## 1. Introduction

Properties, such as solubility, logD, or serum albumin binding have a direct impact on the likelihood of a compound to be successful in clinical trials [1,2]. While measuring these endpoints can be done in a relatively high throughput fashion, it still requires the compounds to be synthesized. In silico prediction tools allow to rank and prioritize compounds before they are synthesized, limiting the amount of experiments performed and thereby saving time and money in drug discovery projects.

Machine learning approaches are typically used to map the structure of compounds to their properties, a method called quantitative structure-activity relationship (QSAR). Common algorithms include multiple linear regression, random forest or support vector machine in combination with circular fingerprints or molecular properties to describe the molecules [3,4,5,6,7]. Water solubility and melting point are two endpoints for which a lot of previous modeling was published [3,4,5,8,9,10,11,12,13].

Membrane affinity and human serum albumin binding, on the other hand, are rarely the subject of QSAR publications. In 2002, Kratochwil and colleagues collected a dataset of 138 compounds with known serum albumin binding and built a partial least squares (PLS) model on top of similarity matrices obtained by comparing pharmacophoric features of the training set. The maximum cross-validation R^2^ was reported at 0.48 [14]. Another study by Ghafourian and Amin [7] used a public dataset of 792 compounds and tried different QSAR methods (linear models, regression trees, random forest, etc.) and reported a boosted tree model with validation R^2^ of 0.65 as best performer.

Since the Merck molecular activity challenge in 2012 [15], it became clear that Deep Learning can increase the performance of QSAR models [16,17], at least when sufficient data is available.

The idea to learn jointly over multiple endpoints, or *multitask learning*, is neither new nor specific to the cheminformatics field. Multitask neural networks share parameters in (some of) their hidden layers between all tasks, forcing the learning of a joint representation of the input that will be useful to all tasks. The main advantages of multitask learning are (i) a regularization effect, as the model has to use the same amount of parameters to learn more tasks; (ii) a transfer learning effect, whereby learning related tasks helps extracting features that are useful in a more general way; and (iii) a dataset augmentation effect, as smaller tasks can be combined together with large tasks avoiding overfitting on the small task [18].

In 2014, Dahl et al. published the first deep multitask approach to classify the bioactivity of compounds in about 20 different assays. They observed an increased ROC AUC performance of 0.04 on average compared to random forest models, with improvements on individual tasks ranging from no improvement to up to +0.17 [16].

In 2016, Kearnes and colleagues [19] published a related work whereby 22 undisclosed ADMET endpoints from Vertex Pharmaceuticals were modeled together in a large multitask neural network. The endpoints had between a few hundred and a few ten thousand data points. The authors showed that single task neural networks would outperform Random Forest AUC performance by 0.05 on average, while combining all endpoints in one model increased the average baseline AUC performance by 0.1.

The previously mentioned studies used pre-established molecular descriptors to encode the chemical structures in a computer-readable way, such as circular fingerprints [20]. Recent progress in handling graph data in neural networks [21,22,23] has been exploited in the cheminformatics field. In 2015, Duvenaud and colleagues proposed an algorithm to learn molecular fingerprints using convolutional networks on a graph representation of the compounds. In this paradigm, atoms correspond to nodes in the graph and bonds are the edges connecting the nodes. The features are learned at the node level, using the adjacency matrix of the graph to communicate information between neighboring nodes [24].

Concurrently to our work, Feinberg et al. published on their approach at Merck to model ADMET properties using a type of graph convolutional networks named PotentialNet [25]. They found great improvement over classical approaches using fingerprints and Random Forest models for many endpoints, including protein plasma binding, solubility, and logD. The average absolute improvement in R^2^ over 31 tasks was 0.14 in temporal splits. PotentialNet is a type of graph convolutional networks that have been designed to predict protein-ligand affinities based in gated graph neural networks. They distinguish different edge types and use a gated recurrent unit to learn information selectively [26].

In this paper, we combine ten different physico-chemical ADMET endpoints into a single multitask graph convolutional regression model. Our graph convolutional networks are simple in that they do not distinguish bond types and do not contain a recurrent unit, but rather follow the Duvenaud algorithm. We show how changing the learning paradigm (from regular machine learning methods to deep learning), the way to describe compounds (from traditional circular fingerprints to end-to-end learned features) and combining endpoints into one model help improve the performance for most of the endpoints.

We also show that adding helper tasks (three endpoints for which no good prediction is required) can help boost the performance on more difficult, smaller endpoints like solubility. Similarly, we show that for very easy and large tasks, combining them into a multitask model does not bring them further predictivity. All validations are performed in varied settings beyond random splits, mimicking real world use cases such as time splits or cluster splits.

## 2. Results and Discussion

### 2.1. Datasets Sizes and Overlaps

**Figure 1:**
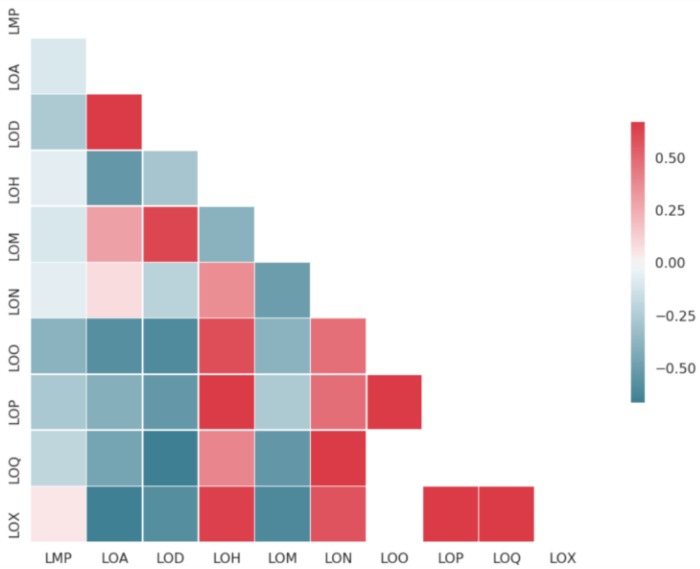
Pearson’s correlation coefficients between pairs of endpoints. When less than 25 compounds were measured in both members of the pairs, no correlation is reported. Endpoint codes are listed in Table 1.

Table 1 reports the different datasets used in this study. The smallest dataset is solubility measured from powder material, while the largest is logD at acidic pH. Figure 1 reports the pairwise correlations between datasets using shared compounds between endpoints. As expected, all solubility endpoints are correlated between them. LogD in acid and neutral pH are also correlated. Human serum albumin binding (LOH) is correlated with solubility, while logD is anticorrelated with solubility. Melting point (LMP) is not very strongly correlated with any other endpoints in this study. Value distributions of the ten endpoints can be found in the Supplementary Materials Figure S2. In total, the datasets together contain 537,443 unique compounds, of which about 79% occur only in one endpoint, 11% are shared between two endpoints, 9% are shared between three. and 1% between four or more. The pairs of endpoints with most overlapping compounds are membrane affinity (LOM) with human serum albumin binding (LOH), LOM with the solubility without assay annotation (LOQ), LOM with the nephelometric solubility assay (LON), logD (LOD) with DMSO solubility (LOO), LON with LOH, and LOQ with LOH.

### 2.2. Performance of Single Task Models

Three types of single task models were built: Random Forest (RF) and fully-connected, feed-forward neural networks (STNN), as well as graph convolutional networks (GCNN). Both RF and STNN are built upon circular fingerprints, while graph convolutional networks learn their feature representation in an end-to-end fashion, starting from the molecular graph and 75 simple atomic descriptors as initial node features.

Table 2 shows the leave-cluster-out cross-validation performance for the different endpoints in different modeling situations. The first three columns correspond to the single task case, where a model is built for each endpoint independently from the others. For all tasks except melting point and solubility from powder, fully connected neural networks greatly outperform Random Forest. On average, R^2^ is improved by 0.06 and Spearman’s rho is improved by 0.05 in the cluster cross-validation setting. These improvements are in line with previous observations [16,19]. The endpoints that are best modeled by the STNN are the two logD, membrane affinity and solubility from nephelometry. These are large tasks (between 64,000 and 230,000 datapoints, Table 1). Solubility from powder and from DMSO not fully dissolved and melting point are the less well predicted endpoints. The two solubility endpoints have the least data and the low performance in a cluster split setting can be explained by overfitting, but melting point is actually one of the largest tasks with 90,000 training examples, so the reason for the poorer performance might have to be found somewhere else. Melting point is notoriously difficult to predict [27] even though the experimental data is very accurate.

Switching from a fixed compound representation (circular fingerprints) to learnt features (graph convolutional networks) allows us to further gain predictive performance in many cases. On average, R^2^ is improved by 0.06 (over STNN) and by 0.12 (over RF) while Spearman’s rho is improved by 0.05 (over STNN) and by 0.29 (over RF) in the cluster cross-validation setting. The only task for which graph convolutional features seem to be detrimental is solubility from powder (LOP) which shows a drop in R^2^. In random split cross-validation though, the performance of the graph convolutional network for LOP is on par with the ones of RF and STNN (see Supplementary information Table S2). We, therefore, assume that the training of graph convolutional networks tends to overfit on smaller training sets. This would explain why the performance in random split appears high (compounds in the test splits are likely similar to compounds in the training set, so the learnt features work well also for the test data) while the performance in cluster splits drops significantly (cluster split cross-validation shows performance in chemical areas that are far away from the training set, where the learnt overfitted features generalize poorly).

It is worth mentioning that intensive hyperparameter selection was not necessary in our case. For Random Forest with ECFC6 fingerprints, we used the default settings from Pipeline Pilot, which is nowadays one of the go-to method for QSAR models in computational molecular design at Bayer. For STNN, a few pyramidal architectures were tested, dropout and input noise were included for controlling overfitting, and the details of batch size, learning rate, etc. were tuned on a cross-validation split for the task melting point only and applied to all other endpoints. As can be seen from Table 2, those parameters seem to perform well on the other ADMET datasets tested, which is something already observed by Ma and colleagues [17].

Note that our final settings follow the guidance provided by the authors: most of our endpoints are log-transformed, we use 4 hidden layers of decreasing sizes with decreasing amounts of dropout and ReLU as activation function. Two main differences are our usage of input noise followed by a tanh transformation to counteract the fact that dropout at input is not appropriate for our sparse fingerprint data and the choice of a bias of –1 for the output layer which we found experimentally to improve performance. In another study, Zhou et al. evaluated different parameters and architectures for single task models for industrial ADME endpoints and found that a pyramidal architecture, dropout and weight decay were beneficial, that models built with ReLU were less sensitive to other hyperparameters, and that regression tasks require smaller learning rates than classification tasks [28]. The graph convolutional STNN settings were also taken as recommended in DeepChem and not further optimized due to the lengthy training process, but the good performance of the trained models shows here again a practical robustness to adjustable parameters.

### 2.3. Performance in Multitask Setting

Since many of the endpoints of interest have some biological relations and actual correlations (Figure 1), we hypothesized that learning all the tasks together would bring further performance improvement. Indeed, by learning simultaneously several tasks, the model has to learn feature representations that are useful for all tasks (regularization aspect) and smaller tasks will benefit from the chemical space coverage of the larger tasks.

We built fully-connected multitask networks and graph convolutional multitask networks (Table 2, last two columns). In the fully connected version (MTNN), the task that sees most improvement is the small solubility from powder endpoint (LOP, 0.29 increase in R^2^ and 0.16 increase in Spearman’s rho). Most of the larger tasks are either not affected or show poorer performance in multitask than in single task approach.

This confirms previous observations that larger tasks are negatively affected by joint training [17]. On the other hand, all solubility endpoints get better predicted. This can be explained by the high correlation between the different solubility assays (Figure 1). The best MTNN model used balanced task weighting when calculating the loss, meaning that tasks with large amount of training data would see their loss down weighted with respect to less represented tasks. One consequence is that the model is allowed to make more errors in the larger tasks, a phenomenon that can be seen when looking at the performance of our largest endpoint, logD in acidic pH (LOA). This could explain the lower performance in MTNN for this particular task (0.08 decrease in R^2^ and 0.03 decrease in Spearman’s rho).

We saw in the single task approach that graph convolutional networks showed higher performance than fully-connected networks, and the same is true in the multitask learning approach: on average, R^2^ increased by 0.17 with respect to the non-convolutional network and Spearman’s rho by 0.09. Comparing the single task with the multitask convolutional networks leads to similar observations as when comparing STNN with MTNN in the non-convolutional setting. The average improvement in performance is 0.14 in R^2^ and 0.06 in Spearman’s rho, but the endpoint-by-endpoint picture is more nuanced. Endpoints like logD (LOD) or melting point (LMP) show no change in performance, while the acidic logD (LOA, our largest task) is negatively impacted in the multitask setting. The tasks benefitting the most from the joint training are the two smaller solubility endpoints (solubility from powder LOP and solubility from DMSO not fully dissolved, LOX). We also notice that the standard deviations of both reported metrics are the smallest for the multitask graph convolutional model, meaning that learning is very stable even across potentially very different cross-validation folds (we report in supplementary Table S1 the standard deviations of the cluster cross-validation results, which in practice contains folds of unequal sizes and difficulty).

### 2.4. Effect of Helper Tasks

Not all endpoints under consideration here are of interest to medicinal chemists, our end users. The nephelometric assay (LON) is not in use anymore and, therefore, the training set contains only historical data. The solubility from DMSO not fully dissolved (LOX) probably contains a lot of artefacts. And finally, the other solubility assay where no assay information was recorded (LOQ) also contains mostly historical data (see Table 1). It means that the actual performance of the models on those three datasets is of little importance, but we included them for completeness and because, in joint training, they might help train the other solubility endpoints (LOO and LOP). We compared the effect of including or not those three helper tasks into the multitask graph convolutional model.

From Table 3, one observes that the endpoints’ performances stay stable without the helper tasks. The two solubility endpoints, which we would assume to benefit most from the helper tasks (recall that these are different solubility assays), show indeed a slightly lower performance in the absence of helper tasks (−0.02 R^2^ for the DMSO solubility and −0.02 Spearman’s rho for the powder solubility). We deduce that adding the helper tasks is not detrimental to the proper learning of the model but will help reaching more accurate predictions in solubility. Beyond considerations on the performance level, one can also argue that adding more related endpoints will also enrich the chemical space covered by the training set, helping the graph convolutions to learn meaningful atom representations and increasing the generalization capability of the model.

### 2.5. General Solubility Equation

The aqueous solubility of a small molecule is linked to its melting point and octanol-water partition coefficient by the general solubility equation (GSE) [29]:

(1) $${\mathrm{logS}}_{w}\;=\;-0.01\;\times \left(\mathrm{LMP}-25\right)-\log\left(K_{\mathrm{ow}}\right)+0.5$$

where logS_w_ is the logarithm of base 10 of the aqueous solubility in mol/L, LMP is the melting point in Celsius degrees, and K_ow_ is the partition coefficient.

We applied this formula to our dataset. In total, 105 compounds had measurements for all three endpoints LOO, LMP, and LOD. The Pearson correlation between the predicted logS_w_ using the GSE with the original 105 LOO data points is 0.75.

We compared this correlation with the one obtained on the cluster split test set by our multitask graph convolutional network. On almost 4000 LOO data points not seen by the model (and in a different chemical space than the training set), the Pearson correlation coefficient between predictions and measurements is 0.81. We also used the model to predict melting point and LogD for these 4000 test datapoints and see whether the model predictions also follow the GSE. For this, we used the predictions of the model for melting point and LogD, obtained the aqueous solubilities according to the Yalkowsky equation and compared these with the predicted LOO. The Pearson correlation is here 0.83, meaning that our model follows globally the GSE model of aqueous solubility without actually being trained on that constraint. Correlations plots can be found in Supplementary Materials Figure S3. We saw from the endpoints correlation matrix (Figure 1) that LogD is clearly anti-correlated with solubility. To check that the GSE property of our network is not simply due to the correlation of logD and solubility in the training data, we also computed the Pearson correlation between the predicted −logD and the predicted solubility for the 4000 test datapoints: this correlation is 0.71, a clear drop in magnitude with respect to the correlation when taking into account both the predicted logD and melting point and following the GSE formula.

### 2.6. Performance in Time Splits

All the results previously commented were obtained by clustering the compounds by structure, then validating the models on left-out clusters of compounds (leave-cluster-out cross-validation). This type of validation shows how well the model generalizes and performs on unseen chemical space. Another way to evaluate models in an industrial setting is to apply time splits. In this approach, all measured data up to a given date are used for training while all recent data are used as a separate test set. We retrained our MTNN graph convolutional model on such a historical subset of our assays, and used all data measured after June 2014 as test set. Table 4 reports the obtained performance on the test sets. Note that, for LOA, no test date could be retrieved so the split is random.

The number of data points for each endpoint vary, as some assays are not often used anymore (melting point, membrane affinity) while others are intensively requested in the course of ongoing drug discovery projects. In terms of performance, we observe slightly lower values in the time split than in the average of the leave-cluster-out cross-validations (R^2^ dropped by 0.06 and Spearman’s rho by 0.03). Still, the performance of our multitask model is solid also in this prospective type of validation. We added the root mean squared error (RMSE) in addition to the usually reported R^2^ and Spearman coefficients for the reader to have a better idea of the typical errors the model is making in time splits. We note that even in such a difficult setting, the model manages an error below one log unit for the two solubility assays of interest, and below half a log unit for the logD predictions. A similar table in the supplementary information shows the results for the *strict time splits* (see Section 3 for more details), where a compound measured in several endpoints can only occur either in training or in test for all endpoints (Table S3). From the relatively robust performance in leave-cluster-out and prospective time split validation, we conclude that a weekly retraining of the model to aggregate newly measured data points is not mandatory in the production phase of the model.

## 3. Materials and Methods

### 3.1. Dataset

In this work, we collected in-house data for the following ADMET endpoints: logD in neutral and acidic pH, solubility (various assay settings), melting point, membrane affinity, and human serum albumin binding (Table 1).

Biological data corresponding to a given assay was preprocessed the following way: when the same compound is measured more than once for that assay, then the average of the measurements is taken as final experimental value. In case a measurement is preceded by an unequal sign (<10 µM) for example), we report either the double of the value (in case the qualifier is ‘>’) or half of the value (in case the qualifier is ‘<’). For human serum albumin binding and membrane affinity, the log_10_ of the experimental value is taken. For melting point and logD, the values are taken as reported in the experiment. For solubility, the reported value in mg/L is first transformed to mol/L then a log_10_ transformation is applied.

**Figure 2:**
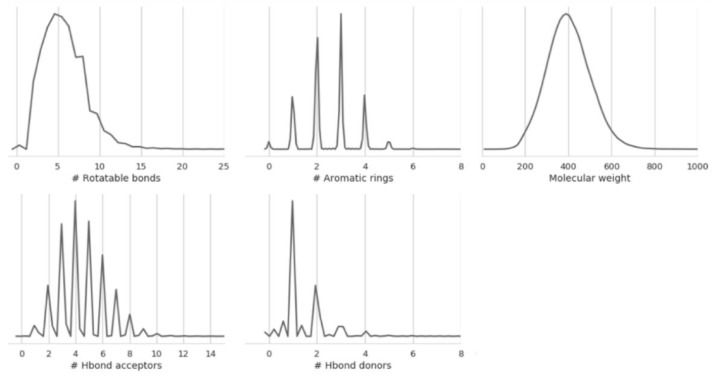
Distribution of molecular properties (number of rotatable bonds, number of aromatic rings, molecular weight, number of H bond acceptors, and the number of H bond donors) in the aggregated dataset containing 537,443 unique molecules tested in at least one of the endpoints of interest.

For the chemical data, we used the Standardize Molecule tool from Pipeline Pilot (Dassault Systèmes BIOVIA, San Diego, CA, USA), selecting “Standardize Charges”, “Keep largest fragment” and “Clear stereo”. Canonical tautomers are generated, then molecules are standardized as neutral by deprotonating bases and protonating acids. Figure 2 shows the distribution of molecular properties in the aggregated dataset containing 537,443 unique compounds.

### 3.2. Model Validation

#### 3.2.1. Data Splits

Models were evaluated in both a cross-validation and a separate test set fashion. Different splitting strategies were applied. We clustered the compounds of the combined datasets using the k-means algorithm (K = 10) and different versions of the ECFC6 fingerprints. Clusters not containing compounds of every task were merged into larger clusters. One cluster was chosen as a test set while the others served as the different folds for the cross-validation set up. Random splits were also performed where compounds would be assigned to a fold randomly, but keeping the folds of the same sizes as those obtained by the clustering procedure and ensuring that each fold contains representatives from each task.

Time splits could not be performed in a cross-validation fashion because we would have needed to find up to 10 measurement dates for which each endpoint would have enough data measured. Instead, we used one temporal split to separate training from test sets. We distinguish two different types of time splits: one where a measurement date is fixed and for each task independently, later measurements are taken as test sets while earlier measurements belong to the training set. This is later referred to as *taskwise time split* as it ignores the fact that a compound measured earlier in one assay might be measured later in another. We also propose a *strict time split* where the training sets are further filtered to remove any compound that would occur in the test set of another task. For one task, logD at acidic pH (LOA), no test dates were available, and compounds were split randomly even in the time split settings.

#### 3.2.2. Performance Measures

The models predict continuous values. The performance of such regression models is evaluated by the coefficient of determination *r^2^* (which measures the concordance between predicted and experimental values) and the Spearman correlation coefficient *rho* (which measures the ranking capabilities of the models). In the case of cross-validation, the individual fold performances are averaged and reported.

### 3.3. Machine Learning Models

#### 3.3.1. Random Forest

Single task models were built using Random Forest regression as implemented in Pipeline Pilot v.18.1 (Dassault Systèmes BIOVIA, San Diego, CA, USA). The input features are extended connectivity fingerprint counts of diameter 6 (hereafter referred to as ECFC6) folded to 1024 or 2048 [20].

#### 3.3.2. Fully-Connected Single Task Network

For the fully connected neural networks, we used a pyramidal architecture with 4 hidden layers (of dimensions 2000, 1000, 500 and 100 respectively) and as input features we used ECFC6 fingerprint counts folded to 1024 or 2048. The activation function used in the hidden units was ReLU, following the observation from Zhou et al. that ReLU seems superior to sigmoid for regression tasks [28]. A decreasing amount of dropout was applied to each layer (50% in the first two hidden layers, 25% in the next, and none in the last hidden layer). Weights were initialized using He’s method [30]. Biases in the hidden layers were initialized to 0 and to −1 for the output layer.

**Figure 3:**
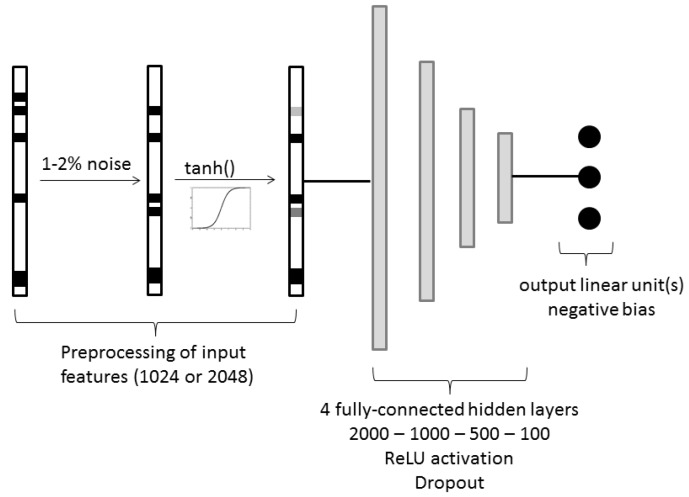
Input feature preprocessing and architecture of the fully connected neural networks. When only one output unit exists, then we talk about single task neural networks (STNN).

Because fingerprints are typically sparse, using dropout on the input features would not have a lot of effect during training. Input noise is used instead to effectively randomly “drop in” chemical features at training time. For this, we generate positive integers by rounding and taking the absolute value from samples of a normal distribution with zero mean and standard deviation of 3 to mimic fingerprint counts. Then, the real inputs are replaced by these noisy fingerprints with a probability p (in our experiments, *p* = 0.01 or *p* = 0.02 worked best). To smoothen out the inputs, we apply the hyperbolic tangent (*tanh*) function directly after the input noise step (Figure 3). The mean squared error is used as a loss function. The models were implemented in Tensorflow version 1.2.1. Hyperparameters, such as hidden layer dimensions, learning rate, weight decay, learning rate scheduling, etc., were optimized on the melting point endpoint and then applied to all other tasks.

#### 3.3.3. Fully-Connected Multitask Network

For the multitask version, the same architecture (four hidden layers of dimensions 2000, 1000, 500, and 100, respectively; see Figure 3) as for the single task networks was used. The input noise probability was reduced to *p* = 0.01 and the learning rate, batch size, learning rate scheduling, number of epochs and weight decay were adjusted using cross-validation in the training set.

To learn in a multitask fashion, the loss function corresponds to a weighted average of individual tasks’ mean squared errors. This means that endpoints with different output ranges (for example, melting points in Celsius degrees, and can reach over 200) could potentially participate differently in the global loss. To avoid this problem, we scale each endpoint values to zero mean and unit standard deviation using standard scaling. Due to the unequal sizes of the different tasks, we explore different ways of averaging the individual task losses: in the “simple” setting, each task receives a weight of 1/N with N being the number of tasks represented in the training minibatch. In the “balanced” setting, tasks with fewer examples are proportionally upweighted compared to tasks with more examples in the minibatch. Missing values (input examples without label for some of the tasks) are ignored and do not participate in the task’s individual losses. If a task does not have any training example in a minibatch then it is ignored and does not participate in the overall loss.

#### 3.3.4. Graph Convolutions

Graph convolutional networks learn node features by propagating features from neighboring nodes and learning affine transformations that will help for the task at hand [23]. In case of molecules, the nodes are the atoms and the edges are the bonds of the molecular graph. A training example is a whole graph and the task is a classification or regression at the graph level. Here, we use the implementation of the Duvenaud algorithm [24] in DeepChem v.1.2.1 [31]. We keep the architecture and hyperparameters suggested by the authors for ADMET predictions, namely:

- 75 input atomic features (see Figure S1 for details);
- two graph convolution steps with a feature dimension of 128 each, with ReLU activation functions; and
- a dense layer with 256 units and ReLU activation functions.

These operations lead to learned continuous atomic features of dimension 256. To make a prediction at the molecule level, the individual atom features have to be aggregated. For this, the feature values are averaged across atoms (mean feature) and the maximum value across atoms is also taken (max feature). These two representations are concatenated and a tanh activation function is applied to give rise to a final molecule representation of size 512.

The learning rate was set to 0.001, and a batch size of 128 was used. The models were trained for 40 epochs. Adam optimization was performed with learning rate decay every 1000 steps.

The same architecture and hyperparameters were used in the multitask setting. Endpoint values were standardized with zero mean and unit standard deviation like in the fully-connected multitask counterpart. The loss is a task-weighted MSE.

## 4. Conclusions

In this work, we built a predictive model for seven ADMET assays corresponding to endpoints of high interest: logD, solubility, melting point, membrane affinity, and human serum albumin binding. Combining all the data available, we were able to apply deep learning methods to learn to predict these endpoints. We showed that, as previously observed, neural networks generally outperform Random Forest in the case of large physicochemical datasets, and that joint training approaches bring further performance improvements at least for the smaller endpoints. Moving away from classical compound representations, we showed that graph convolutional networks are a very powerful method that seems particularly suited for more “physico-chemical” endpoints. The best model, a multitask graph convolutional model with three additional helper tasks, showed very robust performance both in cluster splits and temporal splits. This does not mean that ADMET modeling is a solved problem, since in our experience graph convolutional approaches did not work as well for more complex endpoints like Caco2 permeation or in vitro metabolic stability (validations not shown). Also, multitask modeling is still pretty much a trial-and-error type of work, where it is not clear beforehand which tasks should be combined together nor which hyperparameters would work for a particular task combination. One interesting road to explore would be to extract the learned molecular representation from the last fully-connected layer of the multitask graph convolutional network and use it to try and predict other endpoints in a kind of transfer learning approach. Since our model is trained at predicting general physicochemical properties of small molecules, we can assume that this representation will be useful to predict more complicated endpoints linked to toxicity, environmental safety or target binding.

## Figures and Tables

**Table 1:** ADMET datasets used to train the models.

Endpoint	Code	# Compounds	Data Transformation	Helper Task
LogD (pH7.5)	LOD	76,548	none	no
LogD (pH2.3)	LOA	236,280	none	no
Membrane affinity	LOM	64,506	log_10_	no
Human serum albumin binding	LOH	61,398	log_10_	no
Melting point	LMP	90,589	none	no
Solubility (DMSO)	LOO	38,841	log_10_(mol/L)	no
Solubility (powder)	LOP	2334	log_10_(mol/L)	no
Solubility (nephelometry)	LON	88,301	log_10_(mol/L)	yes
Solubility (DMSO not fully dissolved)	LOX	7392	log_10_(mol/L)	yes
Solubility (no assay annotation)	LOQ	50,016	log_10_(mol/L)	yes

**Table 2:** Performance of different learning algorithm in the ten ADMET endpoints. We report the average of cluster split cross-validation folds (not used for parameter tuning). The best performing method is given in bold (as well as those for which standard deviations overlap, see supplementary Table S1 for standard deviations of the folds).

	Random Forest		STNN ^a^		STNN Graph Conv ^b^		MTNN ^c^		MTNN Graph Conv ^d^	
	R^2^	Spearman	R^2^	Spearman	R^2^	Spearman	R^2^	Spearman	R^2^	Spearman
LOD ^e^	0.63	0.81	0.78	0.89	**0.87**	**0.94**	0.75	0.88	**0.88**	**0.94**
LOA ^f^	0.49	0.76	0.72	0.89	**0.94**	**0.97**	0.64	0.86	0.91	0.96
LOM ^g^	0.43	0.68	0.53	0.75	**0.64**	**0.80**	**0.51**	**0.75**	**0.71**	**0.84**
LOH ^h^	0.39	0.65	0.49	0.73	0.56	0.76	0.49	0.73	**0.65**	**0.82**
LMP ^i^	**0.39**	0.63	0.31	0.66	**0.51**	**0.71**	0.35	0.64	**0.51**	**0.73**
LOO ^j^	0.43	**0.66**	**0.47**	**0.69**	**0.47**	**0.73**	**0.49**	**0.71**	**0.59**	**0.77**
LOP ^k^	**0.09**	0.49	**0.03**	0.48	**−0.17**	**0.59**	**0.32**	**0.64**	**0.56**	**0.76**
LON ^l^	0.50	0.69	0.53	0.74	**0.59**	0.75	0.54	0.73	**0.68**	**0.83**
LOX ^m^	0.33	0.61	0.37	0.64	0.33	0.65	**0.48**	0.72	**0.58**	**0.78**
LOQ ^n^	0.46	0.70	0.51	0.74	**0.58**	0.77	**0.53**	0.75	**0.69**	**0.85**

^a^ single task neural network, ^b^ single task graph convolutional network, ^c^ multitask neural network, ^d^ multitask graph convolutional network, ^e^ logD, ^f^ logD in acidic pH, ^g^ membrane affinity, ^h^ human serum albumin binding, ^i^ melting point, ^j^ solubility from DMSO, ^k^ solubility from powder, ^l^ solubility from nephelometry, ^m^ solubility from DMSO not fully dissolved, ^n^ solubility no assay information.

**Table 3:** Performance of the multitask graph convolutional model without helper tasks. Average of cluster split cross-validation folds. In parenthesis, difference with the results from the multitask graph convolutional model in Table 2.

	R^2^	Spearman
LOD ^a^	0.87 (−0.01)	0.94
LOA ^b^	0.92 (+0.01)	0.96
LOM ^c^	0.71	0.84
LOH ^d^	0.65	0.83 (+0.01)
LMP ^e^	0.52 (+0.01)	0.73
LOO ^f^	0.57 (−0.02)	0.76 (−0.01)
LOP ^g^	0.56	0.74 (−0.02)

^a^ logD, ^b^ logD in acidic pH, ^c^ membrane affinity, ^d^ human serum albumin binding, ^e^ melting point, ^f^ solubility from DMSO, ^g^ solubility from powder.

**Table 4:** Performance of the multitask graph convolutional model in a time split dataset.

	R^2^	Spearman	RMSE	Test Set Size
LOD ^a^	0.87	0.93	0.33	32,794
LOA ^b^	0.91	0.96	0.35	46,481
LOM ^c^	0.69	0.86	0.49	197
LOH ^d^	0.55	0.78	0.61	614
LMP ^e^	0.35	0.59	45 °C	55
LOO ^f^	0.48	0.74	0.90	22,803
LOP ^g^	0.54	0.75	0.84	935

^a^ logD, ^b^ logD in acidic pH (random split), ^c^ membrane affinity, ^d^ human serum albumin binding, ^e^ melting point, ^f^ solubility from DMSO, ^g^ solubility from powder.

## Supplementary Materials

The following are available online at https://www.mdpi.com/1420-3049/25/1/44/s1, Figure S1: Input atomic features for the graph convolutional models, Figure S2: Distribution of experimental values for the ADMET endpoints of interest, Table S1: Standard deviations of cluster split cross-validation folds not used for parameter tuning (complementary to Table 2), Table S2: Performance of the different models in random split cross-validation, Table S3: Performance of the multitask graph convolutional model in the strict time split test set, Figure S3: Correlations between solubility in the data, solubility as deduced from the General Solubility Equation (GSE) and solubility predicted by the model. The code to train multitask graph convolutional networks is available on github: https://github.com/fmonta/mtnngc_admet.
