# Supplementary material for: Modeling Physico-Chemical ADMET Endpoints with Multitask Graph Convolutional Networks
Source: Molecules. 2019 Dec 21;25(1):44. doi: 10.3390/molecules25010044 (PMC6982787; doi:10.3390/molecules25010044)
Supplement: Supplementary file 1 [file molecules-25-00044-s001.pdf]

molecules

MDPI

Supplementary information to "Modeling ADMET data with multitask graph convolutional networks"

The following 75 features are encoded for each atom in the molecules:

- Atomic symbol as one-hot encoding from 44 possible choices
- Degree as one-hot encoding from 11 possible choices (0 to 10)
- Total number of hydrogens as one-hot encoding from 5 possible choices (0 to 4)
- Implicit valence as one-hot encoding from 7 possible choices (0 to 6)
- Formal charge
- Number of radical electrons
- Hybridization as one-hot encoding from 5 possible choices (SP, SP2, SP3, SP3D, SP3D2)
- Whether or not the atom is aromatic

Figure S1. Input atomic features for the graph convolutional models.

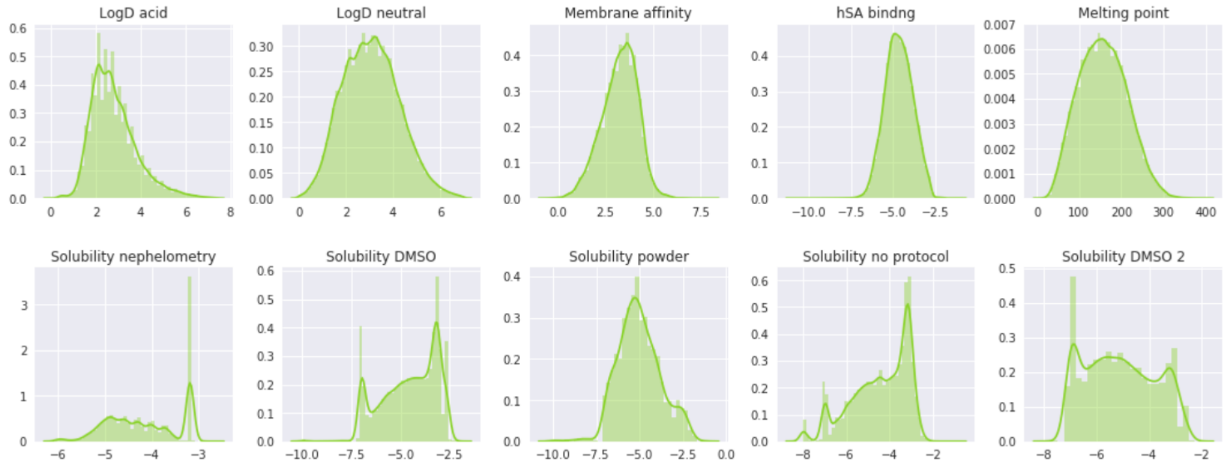

Figure S2. Distribution of experimental values for the ADMET endpoints of interest. Membrane affinity, hSA binding and the solubility endpoints are log-transformed.

molecules

MDPI

Table S1. Standard deviations of cluster split cross-validation folds not used for parameter tuning (complementary to Table 2).

	Random Forest		STNN [a]		STNN GRaph Conv [b]		MTNN [c]		MTNN Graph Conv [d]	
	R^2	Spearman	R^2	Spearman	R^2	Spearman	R^2	Spearman	R^2	Spearman
LOD [e]	0.03	0.02	0.05	0.01	0.02	0.01	0.05	0.01	0.01	0.01
LOA [f]	0.03	0.02	0.05	0.01	0.02	0.01	0.05	0.01	0.00	0.00
LOM [g]	0.10	0.08	0.15	0.07	0.07	0.06	0.20	0.08	0.02	0.01
LOH [h]	0.08	0.05	0.09	0.05	0.05	0.03	0.10	0.04	0.03	0.01
LMP [i]	0.08	0.06	0.08	0.05	0.05	0.04	0.10	0.06	0.04	0.01
LOO [j]	0.06	0.06	0.09	0.09	0.21	0.10	0.08	0.08	0.06	0.08
LOP [k]	0.54	0.15	0.64	0.22	1.08	0.13	0.39	0.15	0.07	0.04
LON [l]	0.07	0.06	0.09	0.05	0.06	0.05	0.09	0.06	0.04	0.02
LOX [m]	0.07	0.04	0.10	0.05	0.11	0.04	0.10	0.03	0.08	0.02
LOQ [n]	0.07	0.05	0.12	0.06	0.08	0.05	0.14	0.06	0.04	0.02

[a] single task neurak network, [b] single task graph convolutional network, [c] multitask neural network, [d] multitask graph convolutional network, [e] logD, [f] logD in acidic pH, [g] membrane affinity, [h] human serum albumin binding, [i] melting point, [j] solubility from DMSO, [k] solubility from powder, [l] solubility from nephelometry, [m] solubility from DMSO not fully dissolved, [n] solubility no assay information.

Table S2. Performance of the different models in random split cross-validation.

	Random Forest		STNN[a]		STNN Graph Conv [b]		MTNN [c]		MTNN Graph Conv [d]	
	R^2	Spearman	R^2	Spearman	R^2	Spearman	R^2	Spearman	R^2	Spearman
LOD [e]	0.81	0.91	0.88	0.94	0.92	0.96	0.84	0.93	0.91	0.96
LOA [f]	0.79	0.90	0.86	0.94	0.94	0.97	0.80	0.92	0.91	0.96
LOM [g]	0.68	0.83	0.71	0.85	0.72	0.84	0.69	0.85	0.70	0.84
LOH [h]	0.65	0.82	0.67	0.84	0.65	0.83	0.67	0.84	0.62	0.83
LMP [i]	0.54	0.73	0.44	0.75	0.56	0.75	0.49	0.74	0.53	0.74
LOO [j]	0.63	0.80	0.65	0.82	0.67	0.82	0.66	0.82	0.68	0.84
LOP [k]	0.52	0.71	0.51	0.72	0.52	0.72	0.63	0.79	0.63	0.79
LON [l]	0.71	0.84	0.71	0.85	0.72	0.85	0.71	0.84	0.69	0.83

LOX[m]	0.57	0.75	0.59	0.77	0.61	0.79	0.68	0.83	0.66	0.82
LOQ[n]	0.66	0.82	0.68	0.84	0.69	0.84	0.68	0.84	0.71	0.85

[a] single task neurak network, [b] single task graph convolutional network, [c] multitask neural network, [d] multitask graph convolutional network, [e] logD, [f] logD in acidic pH, [g] membrane affinity, [h] human serum albumin binding, [i] melting point, [j] solubility from DMSO, [k] solubility from powder, [l] solubility from nephelometry, [m] solubility from DMSO not fully dissolved, [n] solubility no assay information.

Table S3. Performance of the multitask graph convolutional model in the strict time split test set.

	R^2	Spearman	RMSE	Test Set Size
LOD[a]	0.86	0.93	0.42	23 164
LOA[b]	0.90	0.95	0.38	47 250
LOM[c]	0.62	0.80	0.50	199
LOH[d]	0.56	0.74	0.60	646
LMP[e]	0.21	0.47	49°C	55
LOO[f]	0.62	0.80	0.93	8 068
LOP[g]	0.50	0.73	0.81	584

[a] logD, [b] logD in acidic pH, [c] membrane affinity, [d] human serum albumin binding, [e] melting point, [f] solubility from DMSO, [g] solubility from powder.

A. B.

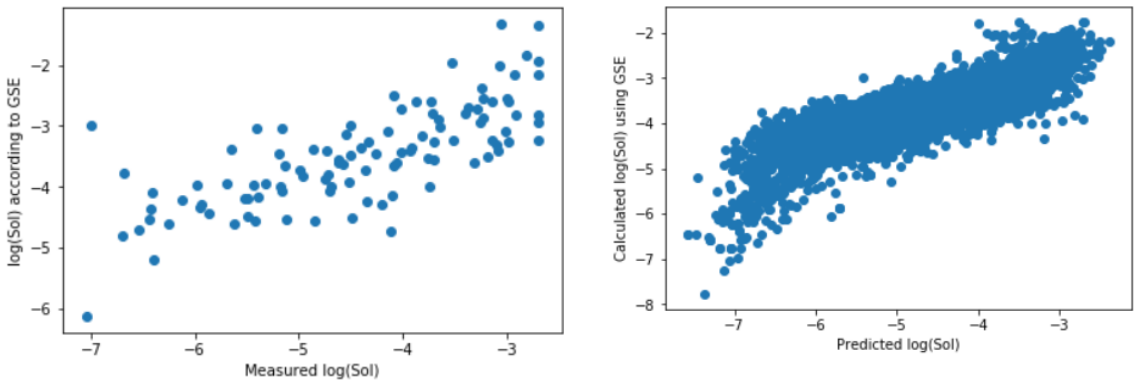

Figure S3. Correlations between solubility in the data, solubility as deduced from the General Solubility Equation (GSE) and solubility predicted by the model. (**A**) Correlation between the measured solubility in DMSO and the calculated solubility according to GSE for compounds having all necessary measurements (LogD, melting point and solubility). (**B**) Correlations between predictions of the multitask graph convolutional model for solubility and calculated solubility according to GSE using the melting point and logD predicted by the model.
